# Low serum sphingosine-1-phospate and its chaperone ApoM associate with retinopathy of prematurity

**DOI:** 10.1016/j.jlr.2026.101030

**Published:** 2026-03-27

**Authors:** Anders K. Nilsson, Ulrika Sjöbom, Mohit B. Panwar, Tove Hellqvist, Zhongjie Fu, Mats X. Andersson, Aldina Pivodic, Lois E.H. Smith, David Ley, Ann Hellström

**Affiliations:** 1 Department of Clinical Neuroscience, Institute of Neuroscience and Physiology, Sahlgrenska Academy, University of Gothenburg, Gothenburg, Sweden; 2 Learning and Leadership for Health Care Professionals, Institute of Health and Care Science at Sahlgrenska Academy, University of Gothenburg, Gothenburg, Sweden; 3 Department of Ophthalmology, Sahlgrenska University Hospital, Region Västra Götaland, Gothenburg, Sweden; 4 Department of Ophthalmology, Boston Children’s Hospital, Harvard Medical School, Boston, MA, USA; 5 Department of Biological and Environmental Sciences, University of Gothenburg, Gothenburg, Sweden; 6 APNC Sweden, Gothenburg, Sweden; 7 Department of Pediatrics, Institute of Clinical Sciences Lund, Lund University and Skane University Hospital, Lund, Sweden

**Keywords:** apolipoprotein M, apolipoproteins, extremely preterm infants, retina, parenteral nutrition, retinopathy of prematurity, sphingosine-1-phosphate, sphingosine phosphate

## Abstract

Retinopathy of prematurity (ROP) is a neurovascular retinal disease affecting extremely preterm infants (<28 weeks’ gestational age), and links between early lipid metabolism and ROP are unclear. We investigated whether the lipid mediator sphingosine-1-phosphate (S1P) and its carrier apolipoprotein M (ApoM) are associated with ROP and parenteral nutrition in preterm infants. In this multicenter cohort, extremely preterm infants were grouped by ROP outcome: no ROP (n = 72) or any ROP (n = 105). Serum was collected at birth and longitudinally to postnatal day 100. S1P was quantified by LC-MS/MS and ApoM by proximity extension assay. Associations between first month mean parenteral fluid intake, S1P, ApoM, and ROP were analyzed using logistic regression; log-normal linear regression was applied to continuous outcomes, adjusting for gestational age and LCPUFA supplementation. Results showed that serum S1P and ApoM were positively correlated (*r* = 0.53, 95% confidence interval [CI] = 0.50–0.56). Higher first-month parenteral fluid intake was associated with lower S1P and ApoM (per 50 ml/kg/day increase, geometric mean ratio = 0.84, 95% CI = 0.79–0.88 for S1P; 0.97, 95% CI = 0.96–0.98 for ApoM; both *P* < 0.001). Higher mean S1P in the first month was associated with reduced odds of any ROP (per 0.1 μmol/l increase, adjusted odds ratio = 0.84; 95% CI = 0.71–0.99; *P* = 0.037). Mean ApoM was associated with ROP only in unadjusted analyses. In conclusion, low S1P and ApoM levels were linked to high parenteral fluid exposure and ROP development, suggesting that infants with high parenteral nutrition requirements may be particularly vulnerable to S1P-ApoM depletion, supporting this pathway as a potential therapeutic target.

Fetal retinal vascularization begins around gestational week 10–15 and is complete by the time of birth (1). In infants born preterm, a significant portion of retinal development occurs ex utero, rendering it vulnerable to perturbations, such as nonphysiological oxygenation and suboptimal nutrition. These disturbances can hinder normal vessel formation and lead to the neurovascular eye disease retinopathy of prematurity (ROP) (2, 3). ROP is the leading cause of preventable childhood blindness worldwide, primarily affecting infants born extremely preterm (<28 weeks' gestational age [GA]). However, more mature preterm infants are also at risk, particularly in low- and middle-income settings (4, 5).

The pathogenesis of ROP follows a biphasic pattern (6, 7). In the initial phase, starting soon after birth or even prenatally, environmental and physiological stressors arrest normal retinal vessel growth. As the infant grows and the metabolic needs of the relatively undervascularized retina increase, hypoxia-inducible factors drive the expression of proangiogenic factors, such as vascular endothelial growth factor (VEGF), thereby reactivating vascular growth. In severe cases, this neovascularization can lead to retinal detachment and blindness if left untreated. Current ROP treatment targets this second, neovascular phase of the disease, typically through laser photocoagulation of the avascular retina and intravitreal anti-VEGF injections. Even though these interventions preserve the central retina and avoid detachment, a history of ROP is associated with long-term visual sequelae (8, 9). Strategies to identify ROP at its initial stage and prevent further progression are urgently needed.

Sphingolipids are a class of lipids with a sphingoid base backbone that serve both structural and signaling functions. Beyond their fundamental roles as components of cell membranes, sphingolipids are increasingly recognized as important regulators of vascular homeostasis and vascular pathology, including endothelial barrier integrity, inflammatory signaling, vascular tone, and angiogenesis (10, 11, 12, 13). In the eye, dysregulated sphingolipid signaling has been implicated in retinal diseases through effects on inflammation, apoptosis, and neovascularization (14, 15, 16).

The bioactive sphingolipid mediator sphingosine-1-phosphate (S1P) plays critical regulatory roles in vascular development (10, 17), endothelial barrier integrity (18), and ocular angiogenesis (12, 19). S1P acts as an intracellular second messenger and as an extracellular signal via a family of five G protein-coupled receptors, designated S1PR_1-5_, of which S1PR_1-3_ are highly expressed in retinal tissues and have been implicated in neurovascular retinal diseases (16), including ROP in animal models (20, 21, 22). During physiological angiogenesis, S1P activates endothelial cell S1PR_1_, inhibiting VEGF-dependent vascular sprouting, and stabilizes the primary vascular network (12, 19, 23). In the blood circulation, S1P is transported by protein chaperones, mainly apolipoprotein M (ApoM) in HDL particles, and to a lesser extent by albumin and ApoA4 (24, 25, 26).

In the murine oxygen-induced retinopathy (OIR) model, which mimics human ROP, ApoM-deficient mice exhibit reduced HDL-bound S1P and increased pathological neovascularization (22). Conversely, ApoM overexpression increases circulating S1P and suppresses neovascularization, an effect mediated via S1PR_1_. Moreover, systemic administration of S1P-loaded ApoM or an S1PR_1_ agonist in OIR suppressed neovascular tuft formation and promoted normal retinal vascularization (22).

In a cohort study of 47 infants born extremely preterm, we found that lower neonatal serum S1P was associated with later development of severe ROP, independently of GA and standardized birth weight (27). Furthermore, prolonged parenteral nutrition has been associated with an increased risk of severe ROP (28) and reduced serum apolipoproteins (A-1, C-2, C-3, and E) (29). The impact of parenteral nutrition on ApoM and its potential role in the pathogenesis of clinical ROP have not been addressed.

This study aimed to confirm the association between circulating S1P and ROP in preterm infants and to further explore whether this association is linked to ApoM levels and parenteral nutrition exposure.

## Materials and methods

### Study design and participants

This cohort study used samples and data from the Mega Donna Mega trial, a multicenter randomized clinical trial aimed at determining the role of enteral supplementation with LCPUFAs (arachidonic acid [AA] and DHA) in infants born <28 weeks GA on severe ROP, other morbidities, and growth (ClinicalTrials.gov identifier: NCT03201588). The trial was conducted at three neonatal intensive care units in Sweden, with recruitment from December 2016 to August 2019. Details about the cohort, study design, and results from primary and secondary analyses have been published (30, 31, 32).

Infants in the intervention arm received a daily dose of a triglyceride oil enriched in AA and DHA (Formulaid™; DSM Nutritional Products, Heerlen, Netherlands). The dose was weight-adjusted to provide 100 mg DHA and 50 mg AA per kg body weight per day. The intervention was started within 3 days after birth and lasted until postmenstrual age 40 weeks. The supplement was administered via nasogastric tube or in the buccal cavity in orally fed infants during feeding. Infants in the control group received only standard nutrition.

A second cohort, consisting of 27 infants born <30 weeks' GA from December 2016 to November 2018, was used to study the relationship between plasma S1P and ROP. The study was conducted at Sahlgrenska University Hospital and at the Sahlgrenska Academy, University of Gothenburg, Sweden; details have been described previously (33). Among these infants, 14 also participated in the Mega Donna Mega trial.

### Collection of morbidity and clinical data

Retinal examinations were performed according to national guidelines, and ROP diagnosis followed the International Classification of Retinopathy of Prematurity (34). ROP was classified as no ROP, mild/moderate ROP (stage 1–2), or severe ROP (stage 3 and/or treated); any ROP included both mild/moderate and severe ROP. Bronchopulmonary dysplasia was defined by mode of respiratory support at 36 weeks postmenstrual age (35); necrotizing enterocolitis was diagnosed by clinical signs and radiological findings (Bell’s stages 2–3) (36); patent ductus arteriosus diagnosis was based on clinical symptoms that required treatment (surgical or pharmacological); intraventricular hemorrhage (IVH) diagnosis was based on cranial ultrasounds performed at postnatal days 3, 7, and 21 and classified according to modified Papile criteria (with severe IVH as IVH stages 3–4) (37); sepsis was diagnosed by clinical symptoms together with a positive blood culture.

Anthropometric and clinical data were extracted from the electronic medical record, and daily nutritional data were registered in the Nutrium™ Software (Nutrium AB, Umeå, Sweden). Standardized birth weight was calculated from the growth charts by Fenton and Kim (38).

### Blood sampling

Blood (0.6 ml) was collected in connection with clinical sampling in serum-separating tubes from the umbilical cord at birth and from peripheral blood on postnatal days 1, 3, 7, 14, and 28 and then every second week until postmenstrual age 36 weeks and at postmenstrual age 40 weeks. The blood was allowed to clot at room temperature for 0.5–2 h before centrifugation at 1,500*g* for 10 min. Serum aliquots were stored at −20°C for up to 1 week before long-term storage at −80°C.

### S1P analysis

To prepare the serum samples, 100 μl methanol containing the internal deuterium-labeled standard D7-S1P (125 nmol/L, Avanti Research, AL) was added to 10 μl infant serum, followed by vigorous mixing and centrifugation at 13,000 *g* for 5 min. The resulting supernatant was transferred to a glass LC vial and stored at −20°C until analysis. Samples were extracted in 22 batches, and in each batch, a quality control (QC) serum sample was extracted using the same method.

The samples were analyzed on an Agilent 1,260 Infinity HPLC system coupled to an Agilent 6,470 triple quadrupole mass spectrometer (Agilent Technologies, Santa Clara, CA) equipped with a Jet Stream ESI source. Chromatographic separation was performed on a ZORBAX Eclipse Plus C18 column (50 × 3.0 mm, 1.8 μm; Agilent) connected to a matching guard column, maintained at 55°C. The mobile phases consisted of (A) 0.1% formic acid in water and (B) 0.1% formic acid in methanol. The flow rate was 0.4 ml/min. The gradient was run as follows: 70% B for 0.1 min, linear increase to 100% B at 5 min, isocratic elution with 100% B until 10 min, followed by 5 min of re-equilibration. A 5 μl sample was injected for analysis. The ESI source was operated in positive mode with the following parameters: a source temperature of 260°C, a nitrogen gas flow at 6 l/min, and a nebulizer pressure of 20 psi. Sheath gas (N_2_) was set to 200°C with a flow rate of 6 l/min. The capillary voltage was 5,000 V, and the nozzle voltage was 500 V. S1P and S1P-d7 were detected using multiple reaction monitoring transitions *m*/*z* 380.3 → 264.5 (S1P) and *m*/*z* 387.3 → 271.1 (S1P-d7). The dwell time for each transition was 80 ms, the fragmentor voltage was 80 V, the collision energy was 27 V, and the cell accelerator voltage was 4 V. A five-point calibration curve (C18-S1P, Avanti Lipids, 0.1–5 μM) was constructed using the same extraction method as applied to infant serum samples, with DDC Mass Spec Gold® Serum (Sigma-Aldrich) as the matrix (*R*^2^ > 0.99). Peak areas were collected in MassHunter Workstation Quantitative Analysis for QQQ (version 10.0; Agilent Technologies), and the S1P concentrations in samples were calculated using a linear regression equation. One sample quantified above the calibration curve was excluded from further analysis. A pool sample was created by taking a small aliquot from multiple infant samples and injected periodically (n = 58) throughout the LC-MS run. The coefficient of variation (CV) of this sample was 4.5% at 0.89 μmol/l. The CV of the batch QC was 6.5% at 0.85 μmol/l.

For analysis of plasma S1P, 50 μl EDTA plasma was aliquoted into cryovials and sent to Metabolon (Metabolon, Inc, Morrisville, NC) for global untargeted metabolomics using a Waters ACQUITY ultraperformance liquid chromatography (UPLC) system coupled to a Thermo Scientific Q Exactive high-resolution/accurate-mass spectrometer with a heated ESI (II) source and Orbitrap mass analyzer operated at 35,000 resolving power, as previously described (39). Briefly, proteins were precipitated with methanol under vigorous shaking for 2 min (Glen Mills GenoGrinder 2000), followed by centrifugation. Samples were briefly dried on a TurboVap® (Zymark) to remove organic solvent and reconstituted in solvents compatible with four chromatographic methods: two reverse-phase (RP)/UPLC-MS/MS methods with positive ion mode ESI, one RP/UPLC-MS/MS method with negative ion mode ESI, and one hydrophilic interaction liquid chromatography/UPLC-MS/MS method with negative ion mode ESI. S1P was quantified from the RP/UPLC-MS/MS data acquired in positive ion mode, using gradient elution from a C18 column (Waters UPLC BEH C18, 2.1 × 100 mm, 1.7 μm) with methanol, acetonitrile, water, 0.05% perfluoropentanoic acid, and 0.01% formic acid. Raw data were extracted, peaks identified, and quality controlled using Metabolon’s proprietary software. Metabolites were identified by comparison to a reference library of purified standards and recurrent unknown features.

### ApoM analysis by targeted proteomics

ApoM was analyzed using the proximity extension assay by Olink (Olink Bioscience, Uppsala, Sweden). Details of the analysis and data preprocessing steps have been described previously (40). Briefly, the serum was gently thawed on ice and centrifuged at 4°C and 1,500 *g* for 20 min. Thereafter, 25 μl was transferred to a 96-well microplate, sealed, frozen at −80°C, and shipped to Olink for analysis. ApoM was analyzed as part of the Target 96 Cardiometabolic (v.3603; OlinkID: OID01221) panel. Additional proteins were analyzed using the Target 96 panels Cardiovascular II (v.5006), Cardiovascular III (v.6113), Development (v.3512), Metabolism (v.3402), and Inflammation (v.3022). After exclusion of duplicate proteins and proteins not meeting QC thresholds, 538 proteins remained for analysis. A pooled reference sample was included on each analyzed 96-well plate, and the CV for ApoM in these samples was 2.7% (n = 17). Protein levels are presented in the arbitrary unit normalized protein expression (NPX), an arbitrary unit on a log2 scale, or as linearized values (2^NPX^). No additional normalization of NPX values was performed before statistical analysis.

### ApoM analysis by ELISA and method comparison

To validate ApoM levels obtained from the Olink analysis and convert relative NPX values to absolute concentrations, and ELISA measurements were compared with the corresponding Olink results. ELISA was performed on both samples reduced with DTT (as described in (41)) and nonreduced samples. As no significant difference was observed between the two conditions (not shown), only results from the nonreduced samples are reported.

Eighteen samples and control samples were selected based on availability and the range of ApoM levels observed in the Olink dataset. These samples were analyzed using the Human ApoM ELISA Kit, NBP2-69839 (Novus Biologicals/Bio-Techne, Minneapolis, MN). Briefly, 10 μl of each sample was diluted 1:1,600 in three sequential steps (1:20, 1:200, and 1:1,600) using the dilution buffer provided with the kit and transferred to a 300 μl 96-well plate. Samples were then analyzed according to the manufacturer’s instructions. Absorbance was measured using a Varioskan Flash plate reader (version 4.00.53; Thermo Fisher Scientific Oy, Vantaa, Finland), and data were processed using SoftMax software (Molecular Devices, San Jose, CA).

To enable conversion of Olink NPX values to absolute ApoM concentrations, Passing-Bablok regression was performed using the mcr package in R. NPX values were treated as the independent variable, and ELISA measurements as the dependent variable. Since Olink data are reported as NPX on a log2 scale, ELISA concentrations were log2-transformed prior to analysis. The coefficient of determination (*R*^2^) was calculated to be 0.806 using ordinary least-squares regression implemented in the base R stats package (Supplemental Fig. S1).

### Ethics

The study was approved by the Regional Ethical Review Board at the University of Gothenburg (ref no.: 303-11, T570-15, 933-16, and T350-18) and the Swedish Ethical Review Authority (ref no.: 2020-02381) and was conducted in accordance with the Declaration of Helsinki (42). Written informed consent to participate was provided by the participants’ parents or legal guardians.

### Statistical analysis

All statistical analyses were performed using R (version 4.4.0) and RStudio (version 2025.09.2, build 418). To assess relationships between S1P, ApoM, and ROP and their modulation by parenteral nutrition, analyses were restricted to the neonatal period, when sampling density was highest, and this represents a critical window for early detection and potential intervention to limit ROP progression.

To summarize each infant’s longitudinal profile of S1P and ApoM during the first month of life, we calculated the area under the curve from postnatal days 0–28 using the trapezoidal rule implemented in the pracma package in R. If the day-28 measurement was missing, a value at day 28 was interpolated using available data up to postnatal day 50. The area under the curve was then divided by 28 to obtain a mean daily value for the first month.

Associations between serum S1P and circulating proteins (including ApoM) were assessed using linear mixed-effects models. For each protein, the NPX value was modeled as the dependent variable, with log2-transformed S1P and postnatal age at sampling as fixed effects and infant ID as a random intercept to account for repeated measurements. Models were fitted separately for each protein, and *P* values for the S1P term were adjusted for multiple testing using the Benjamini-Hochberg false discovery rate method.

To investigate functional patterns among proteins associated with S1P, Gene Ontology enrichment analysis was performed using rank-based gene set enrichment analysis. Proteins were ranked according to the model test statistic for the S1P term, and Gene Ontology Cellular Component was used as the primary ontology.

Continuous variables were described by mean, SD, median, interquartile range (IQR), minimum, and maximum, as applicable, and categorical variables were described by counts and percentages. For comparisons between two groups, Fisher’s exact test was used for dichotomous variables, the Chi-square test for nonordered categorical variables, and the Mann-Whitney *U* test for continuous variables.

For the main regression analyses, first-month mean values of serum S1P and ApoM were used. Mean parenteral fluid intake during the first month was analyzed per 50 ml/kg/day increase. Mean serum S1P and ApoM were analyzed as continuous predictors in logistic regression models for any ROP, with effect estimates reported per 0.1-unit increase. Unadjusted and adjusted logistic regression analyses were performed to evaluate associations between mean parenteral fluid intake in the first month, mean ApoM, mean S1P, and any ROP. Unless otherwise stated, adjusted models included GA at birth and randomized treatment group (AA + DHA or standard care). Results are presented as odds ratios (ORs) with Wald 95% confidence intervals (CIs) and corresponding *P* values.

For continuous, non-normally distributed outcomes, such as first-month mean ApoM and S1P, unadjusted and adjusted log-normal linear regression models were used. These analyses report geometric mean ratios (GMRs) with 95% CIs and *P* values. For plasma S1P analyses in the external cohort, S1P was analyzed in log-relative units, and models were adjusted for GA only. To verify model assumptions, diagnostic plots of residuals were visually inspected to assess linearity and normality.

All tests were two-tailed and conducted at a significance level of 0.05. These analyses represent secondary evaluations from the Mega Donna Mega trial and were conducted in an exploratory manner, intended to generate hypotheses rather than confirm them.

## Results

Two hundred seven infants were randomized in the Mega Donna Mega study, and 178 (86.0%) completed ROP screening. Among the infants who did not complete the study, 28 died (13.5%), and one infant was born with a severe malformation detected after randomization and was thus incorrectly included. Additionally, one infant with oculocerebrorenal syndrome of Lowe, presenting with congenital cataracts, was excluded, as a conclusive ROP diagnosis could not be determined. As a result, 177 infants were included in the final analysis (Table 1, Supplemental Fig. S2). The median (IQR) GA was 25 + 5 (24 + 3 to 26 + 6) weeks + days, and the birth weight was 785 (655–945) g. Regarding ROP diagnosis, 72 infants did not develop ROP; 105 infants developed any ROP; and among these, 55 had mild/moderate ROP (stages 1–2), and 50 had severe ROP (stage 3 and/or treated). As expected, GA and birth weight were lower in infants who developed ROP, whereas the frequency of comorbidities and the use of parenteral nutrition were higher.

**Table 1:** Infant characteristics

Variables	No ROP N = 72	Any ROP N = 105	*P*
GA (weeks)	26.6 ± 0.9	24.9 ± 1.3	<0.0001
Birth weight (g)	901.1 ± 186.5	740.5 ± 183.0	<0.0001
Birth weight SDS	0.08 ± 0.83	0.11 ± 0.82	0.75
Sex, male	42 (58.3%)	58 (55.2%)	0.76
Center			0.0007
1	22 (30.6%)	43 (41.0%)	
2	14 (19.4%)	38 (36.2%)	
3	36 (50.0%)	24 (22.9%)	
Bronchopulmonary dysplasia grade 0 versus 1, 2, or 3	33 (46.5%)	64 (61.5%)	0.06
Necrotizing enterocolitis	3 (4.2%)	11 (10.5%)	0.16
Patent ductus arteriosus			<0.0001
No treatment	51 (71.8%)	30 (29.4%)	
Pharmacological treatment only	17 (23.9%)	52 (51.0%)	
Instrumental	3 (4.2%)	20 (19.6%)	
Missing	1	3	
Severe IVH (grade 3 or 4)	7 (9.7%)	14 (13.3%)	0.64
Total energy intake enterally 1-28 days (kcal/kg)	3,127.8 ± 882.6	2,376.2 ± 973.0	<0.0001
Total energy intake parenterally 1-28 days (kcal/kg)	503.2 ± 419.3	923.1 ± 646.7	<0.0001
Total lipid intake enterally 1-28 days (g/kg)	163.5 ± 54.0	123.4 ± 52.0	<0.0001
Total lipid intake parenterally 1-28 days (g/kg)	15.8 ± 12.8	30.9 ± 22.6	<0.0001

Data are presented as mean (SD) or number (percentage). For tests between two groups with respect to dichotomous variables, Fisher's exact test was used; for categorical variables, Chi-square test was used; and for continuous variables, Mann-Whitney *U* test was used.

### Postnatal changes in S1P and ApoM

**Fig. 1:**
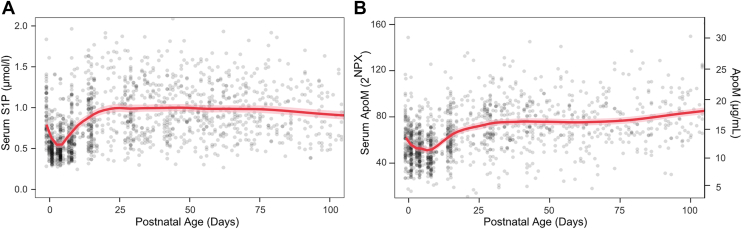
Longitudinal serum levels of S1P and ApoM. Serum S1P (μmol/l) (A) and ApoM (2^NPX^ left *Y*-axis, and μg/ml right *Y*-axis) (B) over the first 100 postnatal days in 177 infants. ApoM concentrations (μg/ml) were derived from NPX values using back transformation based on the assay calibration and should be interpreted as approximate absolute concentrations. Points represent individual measurements, and lines indicate LOESS smooths with shaded areas denoting 95% CIs. LOESS, locally estimated scatterplot smoothing.

Serum S1P and ApoM followed similar trajectories during the first 100 days of life (Fig. 1A, B), and the two were positively correlated over time (*P* < 0.001). The median (IQR) concentration of S1P in cord blood serum was 0.89 (0.72–1.18) μmol/l, decreased rapidly to a nadir of 0.48 (0.41–0.62) μmol/l on day 3, then increased steadily until around week 4 and plateaued at ∼1 μmol/l. There was no correlation between cord blood S1P and GA (Spearman's ρ = 0.09, *P* = 0.944), and the LCPUFA intervention had no apparent effects on S1P or ApoM concentrations (Supplemental Fig. S3).

To estimate absolute ApoM concentrations, a subset of selected samples was analyzed by ELISA and used to convert Olink NPX values to μg/ml (Fig. 1B, right *y*-axis). This analysis indicated that the median serum ApoM concentration over the full study period was around 14 μg/ml.

In an exploratory analysis, we assessed associations between S1P and all proteins included in the larger protein panel. Most proteins (64.6%) were positively associated with S1P (Supplemental Fig. S4), with the largest effect size observed for the cytokine CD40-L. A smaller proportion (15.6%) showed negative associations, including the cardiac hormone B-type natriuretic peptide and NT-pro-B-type natriuretic peptide. Rank-based Gene Ontology enrichment analysis of Cellular Component terms revealed significant enrichment for vesicular, granule-associated, and lysosomal compartments, including vesicle, vesicle lumen, secretory granule lumen, platelet alpha granule, lysosome, and primary lysosome (Supplemental Fig. S5).

### Relationships between S1P, ApoM, and parenteral nutrition

**Fig. 2:**
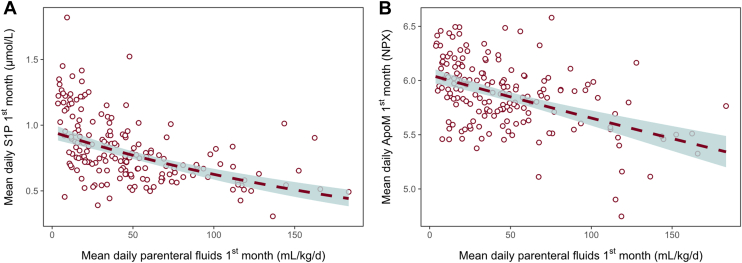
Association between parenteral fluid intake and circulating S1P/ApoM. A: Mean serum S1P in the first month (μmol/l) and (B) mean serum ApoM (NPX) in the first month plotted against mean daily parenteral fluid intake (mL/kg/day). Each dot represents one infant (n = 177). Lines show fitted relationships from linear models with 95% CIs.

Higher parenteral nutrition intake was associated with lower circulating S1P and ApoM. In the unadjusted log-linear model, every 50 ml/kg/day increase in mean parenteral fluid intake during the first month was associated with a 19% lower geometric mean S1P (GMR = 0.81, 95% CI = 0.77–0.85, *P* < 0.001, Fig. 2A) and a 3% lower geometric mean ApoM (GMR = 0.97, 95% CI = 0.96–0.98, *P* < 0.001, Fig. 2B). After adjustment for GA at birth and treatment group, the associations remained similar (S1P, GMR_adj_ = 0.84, 95% CI = 0.79–0.88, *P* < 0.001 and ApoM, GMR_adj_ = 0.97, 95% CI = 0.96–0.98, *P* < 0.001).

### Parenteral fluids, S1P, ApoM, and ROP outcome

**Fig. 3:**
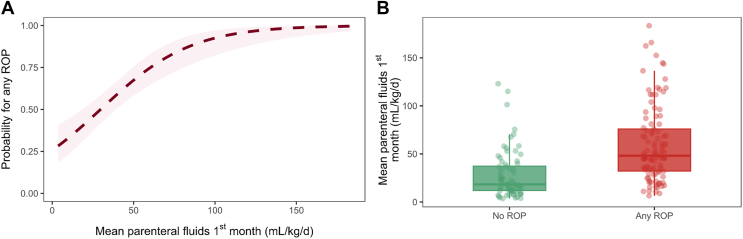
Association between parenteral fluid intake and ROP. A: Predicted probability of developing any ROP according to mean parenteral fluid intake in the first month. The dashed line shows predicted probabilities from a logistic regression model with any ROP as the outcome and mean parenteral fluid intake as a predictor; the shaded area represents the 95% CI. B: Boxplot showing individual mean parenteral fluid intake (g/kg/day) in the first month in infants who did not develop ROP and those who developed any stage of ROP.

Higher mean parenteral fluid intake in the first months was associated with increased odds of ROP (per 50 ml/kg/day increase in parenteral fluid intake, OR = 5.98, 95% CI = 2.92–12.22, *P* < 0.001; OR_adj_ = 2.73, 95% CI = 1.41–5.26, *P* = 0.003) (Fig. 3A). The mean (SD) intake of parenteral fluids in the first month in infants who did not develop ROP was 28.1 (25.1) ml/kg/day compared with 59.5 (39.1) ml/kg/day in infants who developed ROP (Fig. 3B).

**Fig. 4:**
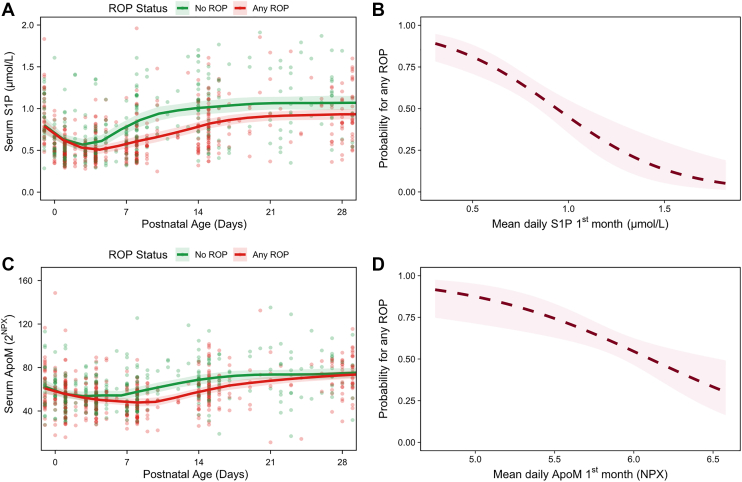
Relationships between serum S1P, ApoM, and ROP outcome. Serum S1P (μmol/l) (A) and ApoM (2^NPX^) (C) over the first 28 postnatal days in 177 infants, stratified by final ROP stage, no ROP versus any ROP. Points represent individual measurements, and lines indicate group-wise LOESS smooths with shaded areas denoting 95% CIs. B and D show the probability of developing any ROP as a function of first-month mean serum levels of S1P and ApoM, respectively (unadjusted models). Lines show fitted relationships from spline models with 95% CIs. LOESS, locally estimated scatterplot smoothing.

In logistic regression, higher mean S1P during the first month was associated with lower odds of any ROP. Each 0.1-unit increase in mean S1P was associated with a 29% reduction in the odds of any ROP (OR = 0.71, 95% CI = 0.62–0.83, *P* < 0.001; OR_adj_ = 0.84, 95% CI = 0.71–0.99, *P* = 0.037) (Fig. 4A, B). Mean ApoM was significant only in unadjusted analyses (per 0.1 unit, OR = 0.84, 95% CI = 0.75–0.94, *P* = 0.002; OR_adj_ = 0.94, 95% CI = 0.83–1.06, *P* = 0.28) (Fig. 4C, D).

### Plasma levels of S1P in relation to ROP

Platelets store S1P and release it upon activation; serum S1P levels are therefore influenced by platelet count (43). Low platelet count/thrombocytopenia has been reported as a risk factor for ROP (44, 45). Thus, to assess whether the lower serum S1P observed in relation to ROP could be explained by low platelet counts, we measured the concentration of S1P in plasma, where the contribution of platelet-derived S1P is small.

**Fig. 5:**
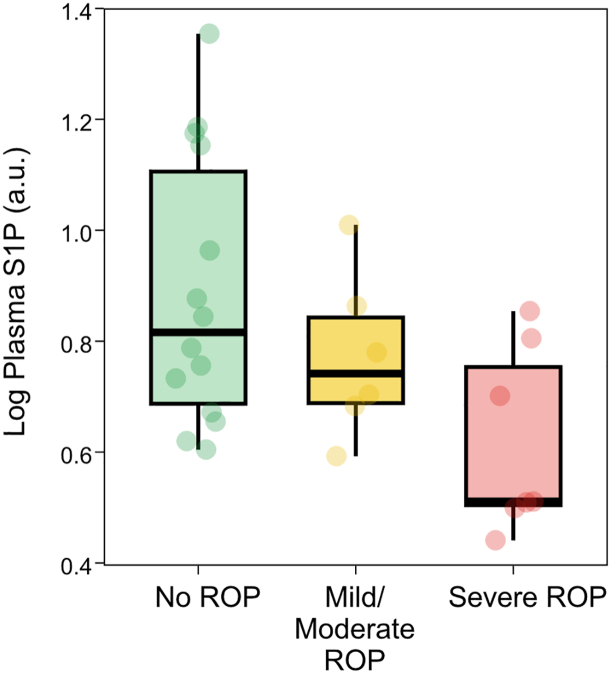
Plasma S1P (log-relative units) at postnatal day 10 stratified by ROP outcome.

To this end, we analyzed S1P in plasma samples collected on day 10 from 27 preterm infants from a cohort partly overlapping with that of the Mega Donna Mega trial (33) (<30 weeks' GA, mean [SD] GA at birth = 26.2 [2.1]). Plasma S1P (log-relative units) was higher in infants who did not develop ROP (n = 14, median [IQR] = 0.82 [0.67–1.15]) compared with those who developed any ROP (n = 13, 0.70 [0.51–0.81]), but the difference was not statistically significant in unadjusted or GA-adjusted analyses (OR *P* = 0.059, OR_GA-adj_ = 0.296). When stratified by ROP severity, comparing no ROP with severe ROP (stage 3 and/or treated, n = 7), the no-ROP group had higher plasma S1P than the severe-ROP group but not significantly after adjustment for GA (OR *P* = 0.047, OR_GA-adj_ *P* = 0.267) (Fig. 5).

## Discussion

This study investigated the relationship between circulating S1P and ROP development in preterm infants. Our analysis focused on the first month of life, when normal retinal angiogenesis is interrupted, i.e., phase 1 ROP, which represents a critical window for interventions that could limit disease progression. Importantly, all analyses were based on samples collected before the development of severe ROP and before the initiation of any ROP treatment. We found that serum S1P was lower in infants who developed ROP and that S1P levels were negatively related to the amount of parenteral nutrition and positively associated with ApoM levels. Together, these findings suggest that high parenteral nutrition administration may reduce circulating ApoM levels and, consequently, S1P levels, thereby contributing to ROP development.

Circulating S1P and ApoM followed similar postnatal patterns, and the two analytes were strongly correlated, supporting ApoM as the principal S1P chaperone in preterm infants. Importantly, this implies that disturbed ApoM production and lipoprotein metabolism can affect S1P levels and signaling.

Parenteral nutrition is essential in extremely preterm infants to supply sufficient energy, protein, essential fatty acids, and micronutrients until enteral feeds are tolerated. However, current formulations are simplified compared with human milk and do not provide many milk-borne nutrients and bioactive components. Most products were developed for adults or older children and are not tailored to the unique metabolic needs of extremely preterm infants. Prolonged reliance on parenteral nutrition is an independent risk factor for ROP (28). Whether this reflects the absence of critical enteral nutrients, the presence of potentially harmful constituents in parenteral solutions, or simply serves as a proxy for overall illness severity remains uncertain.

Based on the negative relationships between S1P/ApoM and exposure to parenteral nutrition, we propose that parenteral nutrition may reduce the HDL-bound ApoM pool, either by lowering circulating HDL or by altering HDL apolipoprotein and subclass composition. Since ApoM is predominantly carried on HDL and contributes to HDL-bound S1P transport, such changes could influence circulating ApoM and S1P levels in parallel. In preterm infants, parenteral nutrition has been reported to influence both HDL apolipoprotein composition and HDL subclass distribution (29, 46), although its specific effect on ApoM has not been addressed. Because standard plasma lipid measurements were not available in our cohort, we cannot distinguish whether the observed associations reflect changes in HDL quantity or composition, ApoM/S1P regulation per se, or shared upstream determinants. A mouse model of parenteral nutrition-associated cholestasis (47) provides a plausible mechanistic link between parenteral nutrition and reduced circulating ApoM. In this model, expression of the nuclear receptor liver receptor homolog-1 is suppressed in the liver (47), and liver receptor homolog-1 is known to induce hepatocyte *APOM* gene transcription and increase ApoM levels (48).

An alternative, not mutually exclusive, mechanism is that the absence of enteral feeding during parenteral nutrition reduces dietary sphingolipid intake and affects infant sphingolipid metabolism. Human milk is rich in sphingolipids, particularly sphingomyelin (49), whereas standard parenteral nutrition contains little to no sphingolipids. In the gut, dietary milk sphingomyelin is hydrolyzed to ceramide and then to sphingosine, of which some is absorbed, reacylated, and used in sphingolipid metabolism (50, 51, 52). Thus, decreased enteral feeding could potentially affect infant S1P levels.

Low ApoM levels have been linked to retinal diseases in adult populations. In the UK Biobank cohort (n = 50,000), plasma ApoM levels were negatively associated with the prevalence and incidence of retinal disorders (53). Furthermore, patients with age-related macular degeneration had significantly lower ApoM levels compared with age-matched controls (14). When the age-related macular degeneration phenotype was recapitulated in a mouse model with impaired retinal cholesterol efflux, overexpression of ApoM ameliorated the phenotype in an S1P- and S1PR_3_-dependent manner (14).

In OIR, genetic loss of S1PR_2_ or sphingosine kinase 2, one of two sphingosine kinases that catalyzes the phosphorylation of sphingosine into sphingosine 1-phosphate, reduces pathological retinal neovascularization (20, 21), and treatment with an S1P-neutralizing antibody similarly suppresses neovascularization (54). In contrast, our clinical data and OIR experiments indicate that lower circulating S1P and loss of ApoM-S1P/endothelial S1PR_1_ signaling is associated with more severe ROP (22, 27). These findings suggest that retinal-intrinsic S1P-S1PR_2_ signaling worsens pathological neovascularization, whereas systemic S1P acting via S1PR_1_ suppressed retinal neovascularization. This compartment- and receptor-specific dichotomy is consistent with the pleiotropic actions of S1P and the distinct roles of its receptors (55). Additionally, the temporal nature of ROP, requiring the establishment of retinal vasculature in phase 1 and the restraint of pathological angiogenesis in phase 2, suggests that S1P may play distinct roles at different disease stages. In this context, it is not surprising that both S1P/S1PR agonists and antagonists have been proposed as therapeutic strategies for retinopathies (21, 22, 54). Based on our findings, supplementation with ApoM (56) or S1PR_1_-specific agonists during the first weeks of life, particularly in infants exposed to high levels of parenteral nutrition, appears to be a promising approach to support early physiologic vascularization. However, substantial work remains to clarify S1P signaling in extremely preterm infants before clinical trials can be undertaken.

Under homeostatic conditions, red blood cells are the dominant source of plasma S1P, with additional contributions from vascular endothelial cells and platelets. Platelets can store S1P and release it upon activation (43), which is why serum S1P levels exceed plasma S1P levels. To ensure that the lower S1P levels observed in the serum from infants who develop ROP are not merely a surrogate for low platelet count, we quantified plasma S1P in a partially independent cohort of premature infants. At postnatal day 10, plasma S1P showed a clear decrease with increasing ROP severity, with the lowest levels in infants who developed higher ROP stages. These results support the conclusion that circulating S1P is genuinely lower in infants who develop ROP.

Consistent with this, exploratory association and enrichment analyses showed that proteins associated with S1P were enriched for terms related to secretory and vesicular compartments, suggesting that circulating S1P is linked to broader pathways of regulated protein release and cellular trafficking rather than only to platelet-derived release during coagulation.

However, the association between plasma S1P and ROP was attenuated after adjustment for GA. This likely reflects the strong collinearity between lower GA and greater exposure to parenteral nutrition, which obscures an independent association of plasma S1P with subsequent ROP severity. Our results showing that birth levels of S1P are unrelated to GA indicate that postnatal events drive GA-associated differences in S1P.

Supporting the specificity of the S1P/ApoM association with ROP, our recent proteome-wide analysis of the same longitudinal Olink dataset showed that only a subset of proteins (109/538), including ApoM, was associated with subsequent severe ROP (57).

In the Mega Donna Mega trial, enteral supplementation with the LCPUFAs AA (ω-6) and DHA (ω-3) reduced the risk of severe ROP by 50% (32). Despite this strong effect, the underlying mechanisms remain poorly understood. In extremely preterm infants, serum phospholipid DHA is positively correlated with S1P (27), and in adults, short-term DHA or docosapentaenoic acid (ω-3) supplementation increases circulating S1P (58), suggesting that the LCPUFA interventions could potentially affect the S1P axis and thereby ROP outcome. However, we did not observe an effect of the intervention on serum S1P. This does not exclude local retinal effects, as DHA can upregulate sphingosine kinase in photoreceptors and stimulate S1P synthesis, implicating tissue-specific modulation that might not be reflected in the circulation (59).

## Strength/limitations

The strengths of this study include controlled repeated blood sampling in a relatively large multicenter cohort of extremely preterm infants and targeted quantification of S1P and ApoM together with prospectively collected detailed nutritional data. The high-frequency sampling over several months allowed the capture of dynamic changes in circulating S1P and ApoM. We found consistency in results across serum and plasma, indicating that the association between serum S1P and ROP is unlikely to be driven solely by platelet-derived S1P. While these observational analyses do not infer causality between circulating S1P/ApoM and ROP, they align well with preclinical studies of the role of S1P in ROP pathology. We also acknowledge that residual confounding may contribute to the observed associations; given the close connection between lower GA, neonatal morbidity, and greater parenteral nutrition exposure, it is not possible to fully disentangle the independent effects of each factor. Finally, higher parenteral fluid volumes may be linked to fluid balance and intravascular volume status at the time of sampling and could influence measured S1P/ApoM concentrations. We cannot distinguish dilution from altered metabolism, and both may contribute to the observed lower levels in infants receiving higher parenteral fluids and subsequently develop ROP.

In conclusion, this study reinforces a role for S1P in ROP pathology and additionally links nutritional practices to circulating S1P via ApoM. Together with preclinical data, these findings support a model in which parenteral nutrition exposure may contribute to lower levels of ApoM and HDL-bound S1P, which could in turn impair endothelial S1PR1 signaling and physiological retinal vascularization in phase 1 ROP. This highlights the ApoM-S1P/S1PR1 axis as a potential modifiable pathway in extremely preterm infants to promote normal retinal vascularization and prevent ROP.

## Data availability

The datasets generated and/or analyzed during the current study are not publicly available because of ethical permits and The General Data Protection Regulation (EU) 2016/679 on the protection of natural persons with regard to the processing of personal data and on the free movement of such data law regulate the availability of personal data, but deidentified data are available from the corresponding author on reasonable request.

## Supplemental data

This article contains supplemental data.

## Conflict of interest

The authors declare that they have no conflicts of interest with the contents of this article.
