# Supplementary material for: Low serum sphingosine-1-phospate and its chaperone ApoM associate with retinopathy of prematurity
Source: J Lipid Res. 2026 Mar 27;67(5):101030. doi: 10.1016/j.jlr.2026.101030 (PMC13138177; doi:10.1016/j.jlr.2026.101030)
Supplement: Supplementary Figures [file mmc1.docx]

**Supplemental material to**

# Low serum sphingosine-1-phospate and its chaperone ApoM associate with retinopathy of prematurity

Anders K Nilsson, Ulrika Sjöbom, Mohit B Panwar, Tove Hellqvist, Zhongjie Fu, Mats X Andersson, Aldina Pivodic, Lois EH Smith, David Ley and Ann Hellström


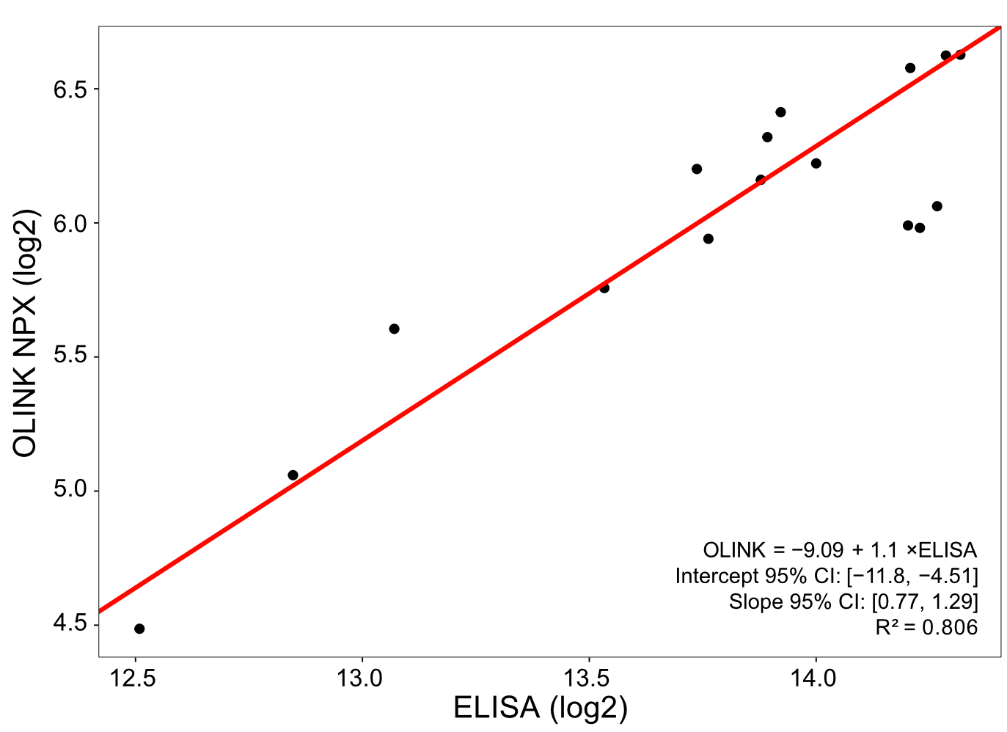


**Figure S1. Method comparison between Olink and ELISA for ApoM.** ApoM levels in eighteen serum samples and control samples were measured by Olink and ELISA. Olink NPX values (y-axis) were compared with log2-transformed ELISA concentrations (x-axis) using Passing–Bablok regression. The fitted regression line is shown in red, together with the estimated intercept and slope with 95% confidence intervals.

**
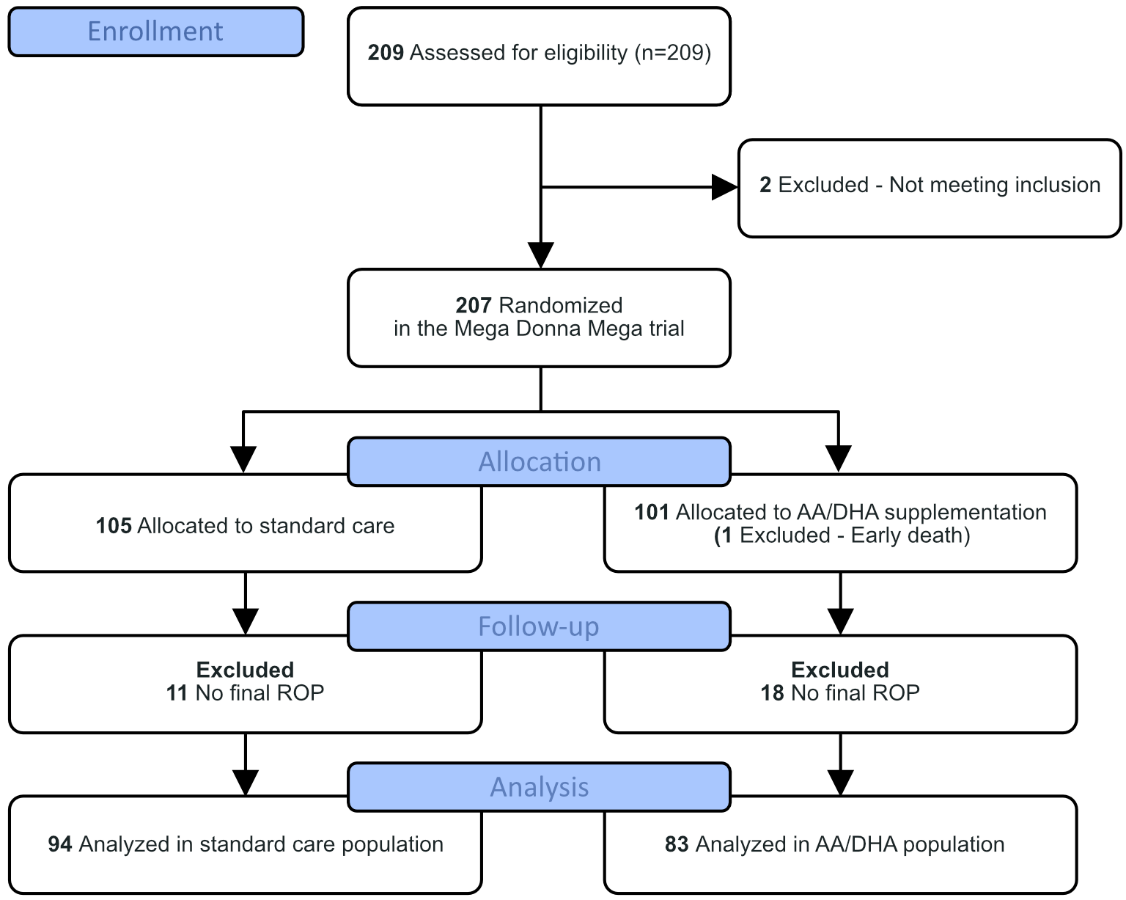
**

**Figure S2.** STROBE flow chart.


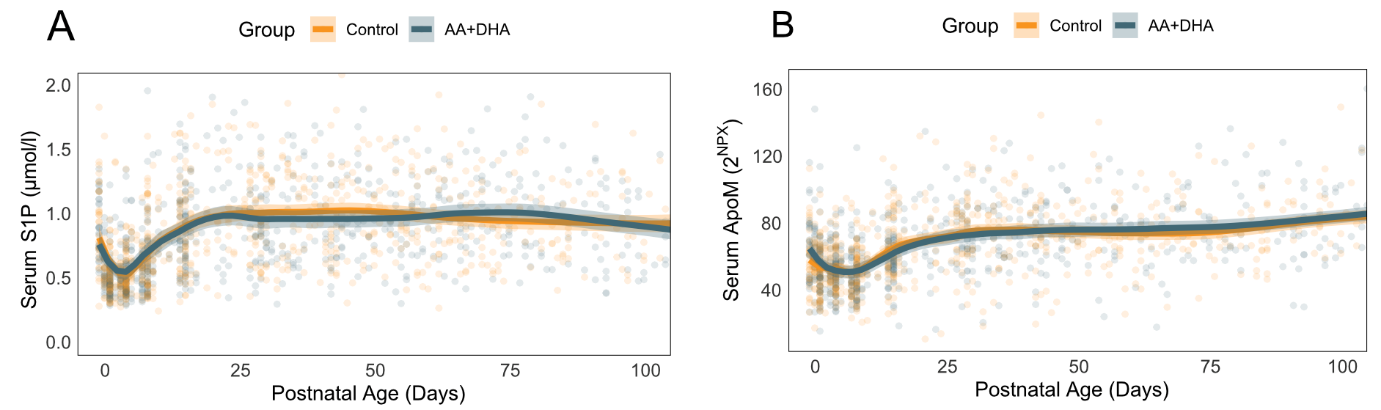
**Figure S3.** **Longitudinal serum levels of S1P and ApoM by randomization group.** Serum S1P (µmol/L) (**A**) and ApoM (2^NPX^) (**B**) over the first 100 postnatal days in 177 infants, stratified by randomization group (Control or AA+DHA supplemented). Points represent individual measurements and lines indicate group-wise LOESS smooths with shaded areas denoting 95% confidence intervals.


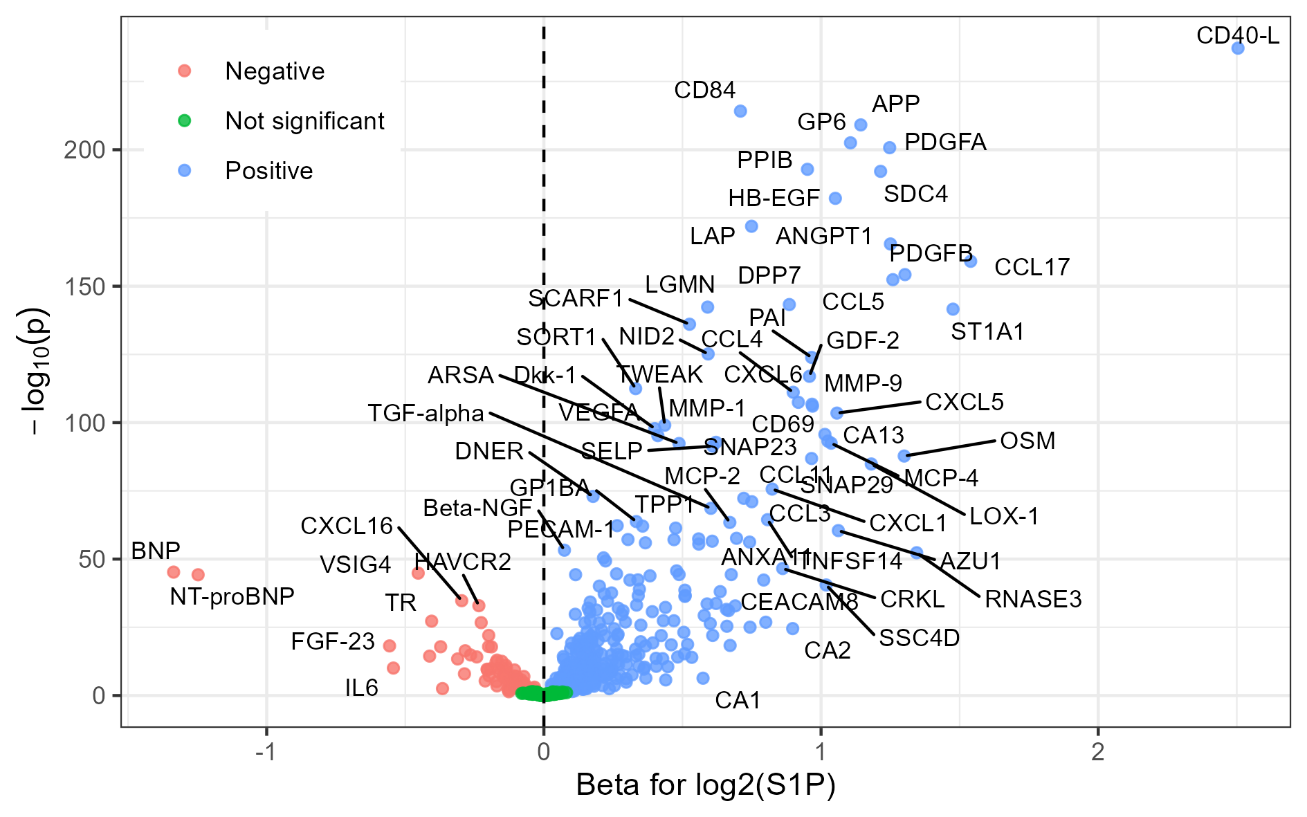


**Figure S4. Exploratory association analysis between circulating S1P and serum proteins.** Volcano plot showing associations between log2-transformed serum S1P and all quantified Olink proteins, excluding ApoM, during the first 100 days of life. Associations were estimated using linear mixed-effects models with postnatal age at sampling as a fixed effect and infant ID as a random intercept to account for repeated measurements. The x-axis shows the regression coefficient for log2(S1P), corresponding to the change in protein level per doubling of S1P, and the y-axis shows –log10(FDR-adjusted p-value). Selected proteins are labeled.


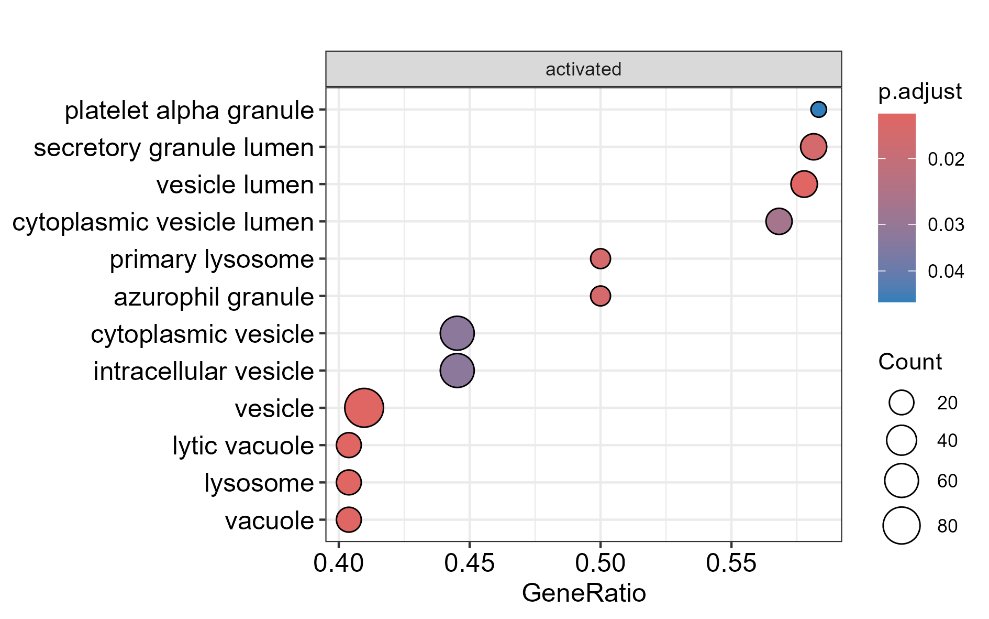


**Figure S5. Gene Ontology enrichment analysis of proteins associated with circulating S1P.** Dot plot showing significantly enriched Gene Ontology Cellular Component terms identified by rank-based gene set enrichment analysis of proteins ranked according to the test statistic from the linear mixed-effects models for association with log2-transformed serum S1P. Significance is shown as FDR-adjusted p values.
